# CD8 ^+^ T‐Cell Deletion Suppressed the Development of Injury‐Induced Experimental Neointimal Hyperplasia in Mice With or Without Chronic Stress

**DOI:** 10.1096/fj.202502380R

**Published:** 2025-09-11

**Authors:** Jingyuan Jin, Meiling Piao, Xianji Piao, Shangzhi Shu, Longguo Zhao, Zhibo Wang, Xueling Yue, Jinshun Piao, Xianglan Jin, Lina Hu, Yongshan Nan, Xian Wu Cheng

**Affiliations:** ^1^ Department of Anesthesiology Yanbian University Hospital Yanji China; ^2^ Jilin Provincial Key Laboratory of Stress and Cardiovascular Disease, Department of Cardiology and Hypertension Yanbian University Hospital Yanji China; ^3^ Department of Cardiology The First Hospital of Jilin University Changchun China; ^4^ School of Public Health Guilin Medical University Guilin China; ^5^ Key Laboratory of Natural Medicines of the Changbai Mountain, Ministry of Education Yanbian University Yanji China; ^6^ Division of Cardiovascular, Department of Internal Medicine, Kyung Hee University Hospital Kyung Hee University Seoul Korea

**Keywords:** CD8^+^ T cell, chronic stress, proliferation, vascular injury, vascular remodeling

## Abstract

Chronic stress exacerbates cardiovascular injury and remodeling. Given the pivotal roles that cytotoxic CD8^+^ T cells play in pathobiology, we investigated potential role(s) of CD8^+^ T cells in stress‐related vascular remodeling in a mouse carotid injury model. Eight‐week‐old male wild‐type (CD8a^+/+^) and CD8a knockout (CD8a^−/−^) mice underwent carotid artery ligation plus cuff placement (L + C) with or without being subjected to chronic stress. At surgery conducted 2 weeks later, L + C alone had significantly promoted carotid neointimal hyperplasia and induced an extensive infiltration of CD8^+^ T cells into injured vascular tissues. Chronic stress further exacerbated neointimal formation and CD8^+^ T‐cell infiltration. Genetically deleting CD8^+^ T cells significantly attenuated neointimal hyperplasia, collagen deposition, and proliferative PCNA‐positive cells and reduced CD68‐positive macrophage infiltration, the expressions of inflammatory genes (AT1R, galectin‐3, MCP‐1, VCAM‐1, ICAM‐1) and extracellular matrix‐remodeling enzyme genes (MMP‐2, MMP‐9, cathepsin S, cathepsin K), and proliferative signaling‐pathway proteins (p‐Akt, p‐p38, p‐mTOR). Data from interferon (IFN)‐γ knockout (IFN‐γ^−/−^) mice administered an IFN‐γ‐neutralizing antibody or an adoptive transfer of CD8^+^ T cells from IFN‐γ^+/+^ mice or IFN‐γ^−/−^ mice further confirmed the CD8^+^ T‐cell deletion‐mediated protective effects against experimental neointimal hyperplasia in response to stress and injury. In vitro vascular smooth muscle cell experiments revealed that the cell migration and invasion abilities and mTOR/Akt signaling were sensitive to 5% stress serum from CD8a^+/+^ mice. CD8^+^ T‐cell deletion thus appears to ameliorate vascular remodeling, suggesting that genetic CD8^+^ T‐cell modification might be a promising therapeutic target for managing proliferative vascular diseases in animals under chronic stress conditions.

## Graphical abstract

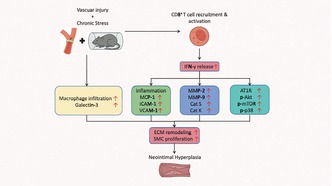

## 1 Introduction

Emerging evidence has spurred a considerable alteration of concepts related to atherosclerosis and has raised questions regarding several associated notions [1]. The initiation and progression of atherosclerotic cardiovascular remodeling involve multiple biological mechanisms including inflammation, oxidative stress, immune activation, cellular proliferation, and apoptosis. An increasing level of attention has recently been paid to the roles of immune cells in atherosclerotic cardiovascular diseases (ACVDs) [2]. As key effector cells of the adaptive immune system, cytotoxic CD8^+^ T cells have been demonstrated to play critical roles in the pathogenesis of vascular injury‐related diseases through adhesion to endothelial cells, the secretion of pro‐inflammatory cytokines, and the induction of local inflammatory microenvironments [3, 4, 5]. Specifically, CD8^+^ T cells exacerbate endothelial dysfunction and promote the proliferation and migration of vascular smooth muscle cells (VSMCs) as well as extracellular matrix remodeling, thereby driving pathological vascular remodeling via direct cytotoxic effects or indirect inflammatory‐immune responses [6]. However, the precise role(s) of CD8^+^ T cells in intravascular intervention‐related neointimal formation and dysfunction have not been established.

Chronic psychological stress (CPS), an exogenous pathological stimulus, significantly increases the risk of ACVDs and other metabolic diseases when sustained over time [7, 8, 9, 10, 11]. CPS activates the hypothalamus‐pituitary–adrenal (HPA) axis and the sympathetic nervous system, subsequently inducing systemic inflammatory responses, which may further exacerbate vascular injury through immune‐cell modulation [12, 13, 14]. Several of our research group's studies demonstrated that chronic stress significantly enhanced high‐fat diet‐induced atherosclerotic plaque development, skeletal muscle injury, hypertension, and renal fibrosis in murine models [15, 16, 17, 18]. It has also been shown that activated CD8^+^ T cells aggravated vascular remodeling and aneurysm formation via inflammatory‐immune mechanisms, although the cells simultaneously impaired ischemic tissue regeneration [19, 20]. We thus hypothesized that chronic stress may exacerbate vascular injury‐induced neointimal hyperplasia by regulating CD8^+^ T‐cell functions.

## 2 Materials and Methods

### 2.1 Animals

Six‐week‐old male wild‐type mice (C57BL/6J, designated as CD8a^+/+^ or interferon [IFN]‐γ^+/+^; C57BL/6J background) were obtained from the Experimental Animal Research Center of Yanbian University (Yanji, Jilin, PR China) and served as the control group. Male CD8a knockout mice (CD8a^−/−19^) and IFN‐γ knockout mice (IFN‐γ^−/−19^) (both on C57BL/6 background) were obtained from Shanghai Biomodel Organism Science & Technology Development Co. (Shanghai, China; protocol no. 2021‐W5‐2174). The mice used in the experiments were 8 weeks old, weighing between 20 and 25 g. All mice were housed in a specific pathogen‐free environment of the Animal Experimental Center of Yanbian University, given ad libitum access to tap water and standard laboratory chow, and maintained under constant conditions (temperature: 23°C ± 1°C, humidity: 50% ± 5%) with a 12‐h dark/light cycle (dark phase starting at 7:00 pm). All experimental protocols were approved by the Yanbian University Animal Ethics Committee (protocol no. YD20231212004).

### 2.2 Data Acquisition

The single‐cell RNA sequencing (scRNA‐seq) dataset analyzed in this study was obtained from the Gene Expression Omnibus (GEO) under accession number GSE244246 (https://www.ncbi.nlm.nih.gov/geo/query/acc.cgi?acc=GSE244246). Carotid artery tissues were harvested from C57BL/6 mice that had been subjected to either carotid artery ligation or a sham operation. For each group, tissues from six individual mice were pooled prior to the RNA extraction and library preparation, resulting in one combined sample per group [21]. Gene expression matrices were downloaded in sparse matrix format (barcodes.tsv.gz, features.tsv.gz, and matrix.mtx.gz) and imported into R using the Seurat package. Quality control procedures were applied to remove low‐quality cells based on the mitochondrial gene percentage, gene count, and unique molecular identifier (UMI) count. Following normalization, integration, and clustering steps, the cells were annotated for the identification of major vascular cell types. Only high‐quality single cells were retained for the downstream analysis.

### 2.3 The Mouse Carotid Artery Injury and Chronic Restraint Stress Model

Mice were anesthetized with isoflurane in an induction chamber (2% in oxygen at a flow rate of 0.5 L/min) and placed supine on a surgical platform at the Animal Center of Yanbian University. The neck region was shaved and sterilized with standard antiseptic procedures. A longitudinal midline incision (~1.5 cm) was made on the anterior neck skin. Under microscopic guidance, the vagus nerve and common carotid artery were carefully dissected and isolated from the surrounding tissues. The right common carotid artery was completely exposed and ligated near its bifurcation with surgical silk. A polyethylene cuff (2 mm long, internal diameter 0.5 mm, external diameter 1 mm) was placed around the artery adjacent to the ligation site [22]. After the surgery, the skin incision was closed, and the mouse was returned to its cage for recovery. The mice in the chronic stress group were subjected daily to either a non‐stress program or the stress program described in an earlier study [23]; the chronic restraint stress program lasted for 14 consecutive days.

### 2.4 Sample Collection

Samples were collected at 14 days after the carotid artery injury and at the completion of the chronic stress protocol. On the indicated time points after the surgery, mice in the stress group underwent 2 h of restraint stress followed by a 1‐h resting period. At the sampling time point, the mice were deeply anesthetized by isoflurane inhalation, and blood was obtained from the cardiac apex immediately after a thoracotomy. The mice were then perfused under physiological pressure with 5 mL of isotonic saline, and the right common carotid artery was carefully dissected and isolated [24].

For a protein analysis by Western blotting, isolated carotid arteries were immediately placed into cryovials and stored at −80°C. For a gene expression analysis, samples were placed in centrifuge tubes containing RNAlater solution, incubated overnight at 4°C, and subsequently transferred to storage at −80°C. For the pathological assessment and immunofluorescence staining, arteries were fixed in 4% paraformaldehyde solution at 4°C for 16 h, embedded in optimal cutting temperature (OCT) compound (Sakura Finetechnical, Tokyo), and stored at −40°C until sectioning [25].

### 2.5 Histological and Immunohistochemical Analyses

Transverse cryosections (5‐μm thickness) were obtained from the carotid arteries at a site 2 mm proximal to the ligation site. Sections were stained using a hematoxylin and eosin (H&E) staining kit (Solarbio Life Sciences, Beijing, China). The perimeters of the vascular lumen, internal elastic lamina (IEL), and external elastic lamina (EEL) were traced on digitized images. The neointimal area was calculated by subtracting the luminal area from the IEL‐defined area, and the medial area was determined by subtracting the IEL‐defined area from the EEL‐defined area [26]. Masson's trichrome staining (Solarbio Life Sciences) was performed to evaluate collagen‐fiber deposition within the vascular wall [25].

For immunohistochemical staining, sections were incubated with primary antibodies including a phycoerythrin (PE)‐conjugated mouse monoclonal anti‐CD68 antibody (1:40; Chemicon International, Temecula, CA, USA), a mouse monoclonal anti‐CD8a antibody (1:100, cat. no. 553032, BD Pharmingen, San Diego, CA), and a mouse monoclonal anti‐proliferating cell nuclear antigen (PCNA) antibody (NA03; Merck Millipore, Darmstadt, Germany). After being washed with phosphate‐buffered saline (PBS), the sections were incubated with a secondary antibody against mouse IgG (1:200, Abcam, Cambridge, MA) following the manufacturer's instructions. We also performed double immunofluorescence using mouse monoclonal anti‐CD8a and rabbit monoclonal anti‐IFN‐γ (1:200, cat. no. #8455, Cell Signaling Technology, Beverly, MA) to identify the cell source of IFN‐γ in the carotid arteries of control, injured alone, and injury+stressed mice.

Signals were visualized with the use of an EVOS microscope (Invitrogen, Carlsbad, CA), and the images were analyzed with ImageJ software. For a quantitative analysis, five randomly selected fields per section were captured at 40× magnification. The numbers of CD8a‐positive, CD68‐positive, and PCNA‐positive cells were counted, and the average number per animal was calculated for the statistical analysis [19].

### 2.6 Western Blot Analysis

Total protein was extracted from carotid artery tissues of mice from the various experimental groups and VSMCs in RIPA lysis buffer containing protease and phosphatase inhibitors [27]. The concentrations of total protein were analyzed with a BCA Protein Assay Kit (Solarbio Life Sciences). Each sample of total protein (40 μg) was separated by sodium dodecyl sulfate–polyacrylamide gel electrophoresis (SDS‐PAGE) and then blotted onto polyvinylidene fluoride (PVDF) membranes.

Next, the membranes were blocked in 5% skim milk or 5% bovine serum albumin (BSA) for 1 h and subsequently incubated overnight at 4°C with primary antibodies against phosphorylated mTOR (p‐mTOR, cat. no. #2971), total mTOR (cat. no. #4517), phosphorylated p38 MAPK (p‐p38 MAPK, cat. no. #4511), total p38 MAPK (cat. no. #9212), phosphorylated Akt (p‐Akt Ser473, cat. no. #4060), total Akt (cat. no. #2967), and GAPDH (cat. no. #5174) (all from Cell Signaling Technology, Beverly, MA; 1:1000 dilution); anti‐angiotensin II type 1 receptor (AT1R, cat. no. ab124505), and anti‐galectin‐3 antibody (cat. no. ab209344) (each from Abcam; 1:1000 dilution).

Following membrane incubation with the secondary antibodies at dilutions ranging from 1:5000 to 1:10,000, molecular targets were visualized with the use of a chemiluminescent substrate kit (Merck Millipore) [28]. The protein expression levels were quantified by densitometry and normalized to the internal standard glyceraldehyde 3‐phosphate dehydrogenase (GAPDH) levels.

### 2.7 Gene Expression Assay

Total RNA extraction and gene expression measurements were performed as described [29]. In brief, after RNA isolation, the total RNA was reverse‐transcribed into complementary DNA (cDNA) with the use of a transcription kit (Zomanbio, Beijing, China) according to the manufacturer's guidelines. A real‐time quantitative polymerase chain reaction (qPCR) analysis was performed with an ABI 7300 Real‐Time PCR system (Applied Biosystems, Foster City, CA) to evaluate the targeted gene expression levels of vascular cell adhesion molecule‐1 (VCAM‐1), monocyte chemotactic protein‐1 (MCP‐1), intercellular adhesion molecule‐1 (ICAM‐1), cathepsin S (CatS), cathepsin K (CatK), matrix metalloproteinase‐2 (MMP‐2), and matrix metalloproteinase‐9 (MMP‐9). These RNA expressions were normalized by GAPDH mRNA as an internal control.

### 2.8 The IFN‐γ Neutralization Experiment in the Arterial Injury Model

After the establishment of the arterial injury model, mice received an intraperitoneal injection of a neutralizing antibody against IFN‐γ (0.5 mg/kg, clone R4‐6A2, Thermo Fisher Scientific, San Jose, CA) or a mouse isotype control IgG (0.5 mg/kg, cat. no. 02‐6502, Thermo Fisher Scientific) on Days 0, 3, 5, and 10 after surgery and then subjected to the sampling [30].

### 2.9 CD8 ^+^ T‐Cell Isolation and Adoptive Transfer

CD8^+^ T cells were isolated from the spleens of male 7‐week‐stressed IFN‐γ^+/+^ and IFN‐γ^−/−^ mice with the use of the CD8a^+^ T cell isolation kit (cat# 130‐104‐075, Miltenyi Biotec, Bergisch Gladbach, Germany) [19]. The purity of the isolated cells consistently exceeded 95%. After the cells' resuspension in 100 μL of PBS, a total of 1 × 10^7^ CD8a^+^ T cells were intravenously infused via the tail vein into CD8^−/−^ recipient mice on Days 1 and 5 after the mice had undergone the carotid artery injury surgery [19].

### 2.10 Cell Culture and Treatments

Mouse aortic VSMCs were purchased from Fuheng Biotechnology Co. (Shanghai, China), and passages 6–8 were used for the experiments. The VSMCs were cultured and expanded in Dulbecco's modified eagle medium (DMEM) supplemented with 10% fetal bovine serum (FBS) at 37°C in a humidified atmosphere containing 5% CO_2_ and 95% air. The VSMCs were then seeded into six‐well plates at a density of 2 × 10^5^ cells per well. Upon reaching approximately 80% confluence, the cells were serum‐starved for 6 h and then treated with 5% non‐stress serum (NS‐serum) or 5% 7‐week‐stressed serum (S‐serum) for another 24 h. After this treatment, the cells were harvested for a western blot analysis.

### 2.11 Cell Migration and Invasion Assays

Cell migration and invasion assays were performed using Transwell chambers (Costar, Cambridge, MA) with polycarbonate membranes with 5‐μm pores [22]. Briefly, the inner‐chamber membrane was precoated with 50 μL of Matrigel solution (50 g/mL, Becton Dickinson, Lincoln Park, NJ) at 4°C for 6 h to avoid polymerization and then washed with PBS for a cell migration assay. The upper surface of the membrane was uniformly coated with 20 μL of the Matrigel solution (0.7 mg/mL) and incubated at 37°C for approximately 0.5 h to allow gelation for a cell invasion assay.

Next, 600 μL of 5% NS‐serum/DMEM or 5% S‐serum was added to the lower chamber of a 24‐well plate. The Transwell inserts were placed into the wells, and then 100 μL of cell suspension (1 × 10^4^ cells/mL for the cell migration assay; 2 × 10^4^ cells/mL for the cell invasion assay) was added to the upper chamber. The plates were incubated at 37°C in a humidified incubator with 5% CO₂ for 6 h (migration) or 24 h (invasion). The cells that had invaded or migrated through the membrane were fixed, stained, and counted in 5–7 randomly selected fields per insert under a light microscope at 200× magnification [22].

### 2.12 Statistical Analyses

All data are presented as the mean ± standard error of the mean (SEM). The statistical analyses were performed after the assessment of the data's distribution. Pairs of groups were compared with Student's *t*‐test, and three or more groups were compared with a one‐way analysis of variance (ANOVA) followed by Tukey's post hoc test. All statistical analyses were conducted using GraphPad Prism ver. 8.0.2 software. A probability (*p*)‐value < 0.05 was considered significant. All morphological evaluations were performed independently by two blinded observers, and the average values were used for analysis.

## 3 Results

### 3.1 The Single‐Cell Landscape of the Ligation‐Induced Vascular Remodeling

**FIGURE 1:**
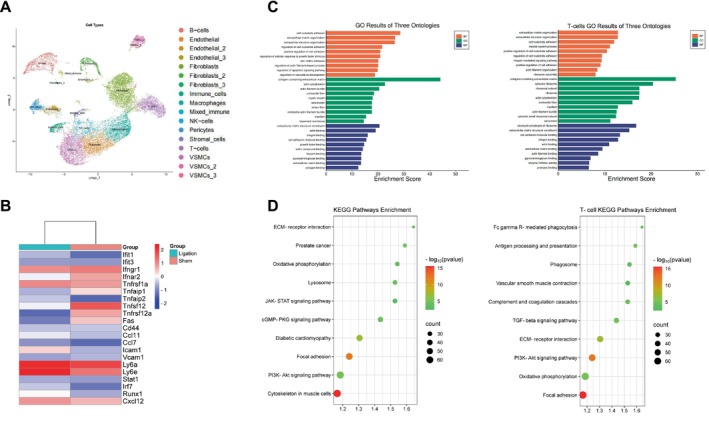
The single‐cell transcriptomic landscape of mouse carotid arteries at 14 days after ligation. (A) The UMAP (uniform manifold approximation and projection) algorithm delineated 18 transcriptionally distinct clusters encompassing both structural and immune cell populations. (B) The results of a heatmap analysis of the 20 differentially expressed genes (DEGs) between ligation and sham groups (|log₂FC| > 1, *q* < 0.05) revealed significant upregulations of interferon (IFN)‐stimulated transcripts (Ifit1/3, Stat1, and Irf7) and adhesion/chemotactic molecules (VCAM1, ICAM1, Ccl7, Cxcl12). (C) Gene ontology enrichment demonstrated that both global DEGs and T cell‐specific DEGs were significantly enriched in the extracellular matrix (ECM) organization and leukocyte migration; the T‐cell‐specific DEGs also showed enrichment in immune effector regulation pathways. (D) A KEGG pathway analysis revealed distinct signatures: Global DEGs were enriched mainly in ECM–receptor interaction, focal adhesion, and PI3K/Akt signaling pathways, whereas the T‐cell‐specific DEGs were associated with FcγR‐mediated phagocytosis, antigen processing/presentation, and vascular smooth muscle contraction. The bubble size represents the gene count, and color denotes the –log₁₀(*p*‐value).

The single‐cell RNA sequencing analysis of pooled sham and ligated carotid arteries revealed 18 transcriptionally distinct cell clusters, with T cells localized in spatial proximity to endothelial cell and VSMC clusters (Figure 1A). The ligation injury induced a prominent IFN signature and enhanced the capacity for leukocyte recruitment, as evidenced by the upregulations of Ifit1/3, Stat1, VCAM1, ICAM1, and Ccl7/Cxcl12 (Figure 1B). The functional enrichment of whole‐tissue differentially expressed genes (DEGs) underscored the presence of extracellular matrix (ECM) remodeling, focal adhesion, and proliferation‐related PI3K/Akt activation, whereas T‐cell‐specific DEGs further mapped to FcγR were observed to mediate phagocytosis, antigen presentation, and VMSC contraction pathways (Figure 1C,D).

These findings established that the carotid ligation engaged the IFN ECM‐PI3K/Akt signaling axis that simultaneously promotes vascular matrix remodeling and multifunctional CD8^+^ T‐cell effector functional reprogramming. This synergistic interaction explains the priming of the injured vessel wall for exacerbated neointimal hyperplasia under chronic stress.

### 3.2 CD8 ^+^ T‐Cell Deletion Mitigated the Injury‐Related Neointimal Hyperplasia, Accompanied by a Reduction of Inflammation

**FIGURE 2:**
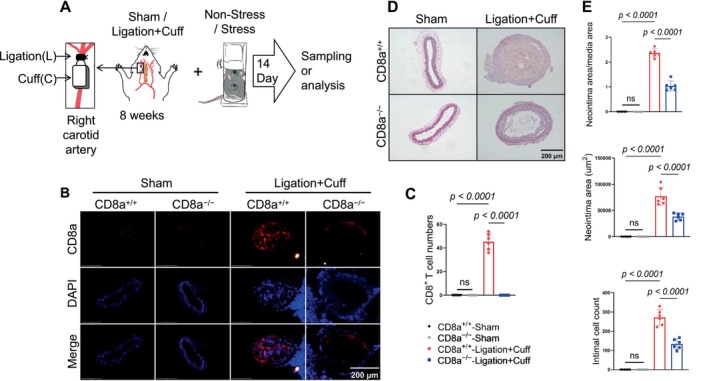
CD8^+^ T‐cell deletion mitigated injury‐related neointimal hyperplasia in mice. (A)‐ Experimental design. Eightweekold male CD8a^+/+^ and CD8a^−/−^‐mice were randomly subjected to a sham operation (Sham) or a carotid artery ligation+cuff (L + C) operation, followed by 14 days of either nonstress (NonStress) or chronic stress (Stress) prior to tissue collection and analysis. (B) Representative immunostaining and quantitative data show the infiltrated CD8a^+^ macrophages (*red*) with DAPI nuclear counterstain (*blue*). Scale bar: 200 μm. (D, E) Representative hematoxylin and eosin (H&E) and quantitative data show the neointima/media area ratio, neointimal area, and intimal cell number (*n* = 6 per group). Data are mean ± SEM (*n* = 6/group). The significance of differences among the groups was analyzed by a one‐way ANOVA (C, D). NS, not significant.

To determine the role of CD8^+^ T cells in vascular remodeling after injury, we established a carotid artery Ligation plus Cuff placement (L + C) model in CD8a^+/+^ and CD8a^−/−^ mice (Figure 2A). Fourteen days after the surgery, the carotid arteries of the mice were harvested for histological and molecular analyses. The immunofluorescence staining analyses revealed a substantial infiltration of CD8^+^ T cells in the injured arteries of the CD8a^+/+^ mice but not in those of the CD8a^−/−^ mice (Figure 2B,C), which suggests that the vascular injury induces CD8^+^ T‐cell recruitment.

**FIGURE 3:**
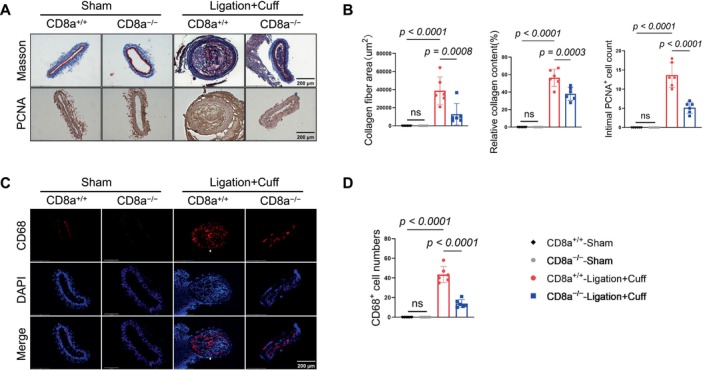
CD8^+^ T‐cell deficiency attenuated the injured vascular collagen deposition, cell proliferation, and macrophage infiltration. CD8a^+/+^ and CD8a^−/−^ mice were subjected to a sham or L + C operation respectively and were sampled 14 days later. (A) Representative Masson's trichrome and PCNA immunostaining images of collagen deposition and PCNA^+^ cells in the sham and L + C injured carotid arteries of mice of both genotypes. Scale bar: 200 μm. (B) The results of the quantitative analysis of collagen deposition, including the collagen fiber area (*left*), relative collagen content (*middle*), and number of PCNA^+^ cells in the injured arteries of the four experimental groups. (C, D) Representative immunofluorescence images and quantitative data for the numbers of infiltrated CD68^+^ macrophages (*red*) in the carotid arteries of the four experimental groups. Scale bar: 200 μm. (D) The quantification of CD68^+^ cell numbers per section. Data are mean ± SEM (*n* = 6 for each group). Significance was assessed by a one‐way ANOVA followed by Tukey's post hoc tests (B, D).

The quantitative data from the H&E staining showed that the CD8^+^ T‐cell deletion significantly reduced the neointima/media area ratio, the neointimal area, and the intimal cell numbers (Figure 2D,E). Masson's trichrome staining indicated pronounced collagen deposition in the injured vessels of the CD8a^+/+^ mice; this deposition was notably suppressed in the CD8a^−/−^ mice (Figure 3A upper panels, Figure 3B). Moreover, the CD8a^−/−^ mice had lower numbers of proliferating PCNA^+^ cells and inflammatory CD68^+^ cells in the injured carotid arteries compared to the CD8a^−/−^ mice (Figure 3A lower panels, Figure 3B−D).

**FIGURE 4:**
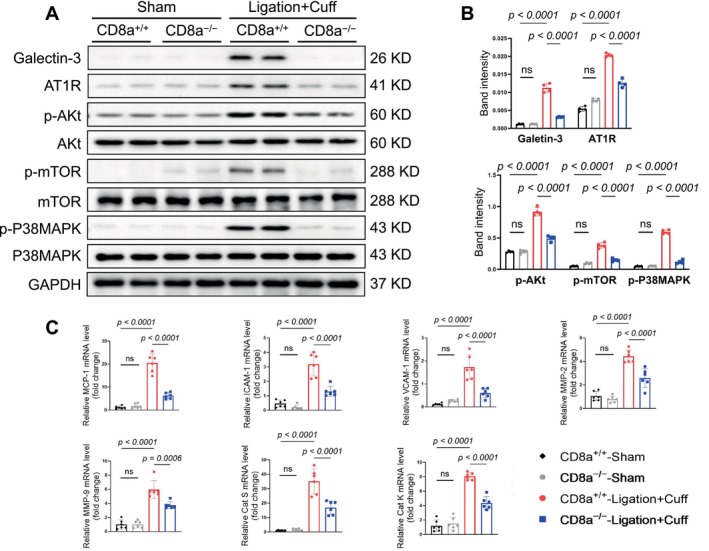
CD8^+^ T‐cell deficiency reduced the investigated molecular alterations in response to the L + C injury. (A, B) Representative western blot images and combined quantitative data for the levels of galectin3, AT1R, p‐Akt, pmTOR, and pP38MAPK in the carotid arteries of CD8a^+/+^ and CD8a^−/−^ mice subjected to the sham or L + C double injury. (C) The quantitative polymerase chain reaction (qPCR) analysis revealed alterations in the levels of MCP1, ICAM1, VCAM1, MMP2, MMP9, cathepsin S, and cathepsin K in injured arteries of the mice in the four experimental groups. Data are mean ± SEM (*n* = 6 for each group). Significance was assessed by a one‐way ANOVA followed by Tukey's post hoc tests (B, C).

To explore the related molecular mechanisms, we conducted a western blot analysis to evaluate the targeted molecules' changes in the non‐injured and injured carotid arteries. As anticipated, CD8a^−/−^ lowered the levels of inflammation‐related proteins (galectin‐3 and AT1R) and proliferation‐related proteins (p‐Akt, p‐mTOR, and p‐p38MAPK) in the injured carotid arteries (Figure 4A,B). The quantitative RT‐PCR analyses yielded the same conclusion regarding the expressions of inflammatory cytokine genes (MCP‐1, ICAM‐1, VCAM‐1) and proteolytic enzyme genes (MMP‐2, MMP‐9, cathepsin S, cathepsin K) in the CD8a^−/−^ mice (Figure 4C). CD8^+^ T cells thus appear to modulate the neointimal hyperplasia that occurs after vascular injury by promoting inflammation, cell proliferation, and ECM remodeling.

### 3.3 Chronic Stress Promoted Neointimal Hyperplasia Following Vascular Injury

**FIGURE 5:**
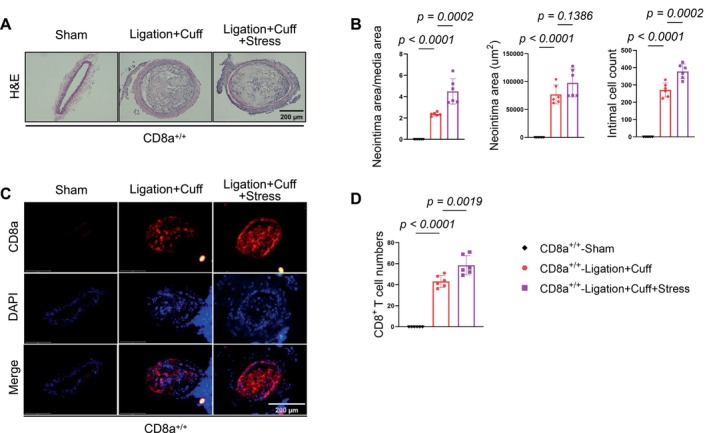
Chronic stress enhanced injury‐reduced CD8^+^ T‐cell infiltration and neointimal formation in the CD8a^+/+^ mice. The CD8a^+/+^ mice underwent a sham operation, L + C injury alone, or stress plus the L + C injury for 2 weeks and were then subjected to sampling for the histological analysis. (A, B) Representative H&E staining images and quantitative data showing the neointima/media area ratio (*left*), neointimal area (*middle*), and intimal cell counts (*right*). (C, D) Representative immunofluorescence images and quantitative data provide the numbers of infiltrated CD8a cells (*red*) in carotid artery cross‐sections of three experimental groups. Scale bar: 200 μm. Data are mean ± SEM (*n* = 6 for each group). Significance was assessed by a one‐way ANOVA followed by Tukey's post hoc tests (B, D).

**FIGURE 6:**
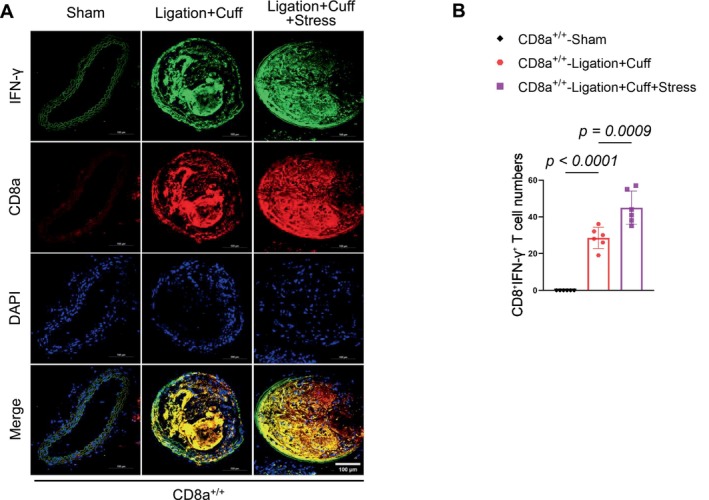
Stress accelerated IFN‐γ^+/+^CD8^+^ T‐cell infiltration into the injured carotid artery tissues of CD8a^+/+^ mice. Double immunofluorescence used anti‐CD8a (red) and anti‐INF‐γ (green) antibodies was applied to identify the IFN‐γ^+/+^CD8^+^ T cells in the three experimental groups. (A, B) Representative double immunofluorescence and quantitative data show the numbers of IFN‐γ^+/+^CD8^+^ T cells in the uninjured and injured carotid artery tissues of the three experimental groups. Scale bar: 100 μm. Data are mean ± SEM (*n* = 6 for each group). Significance was assessed by a one‐way ANOVA followed by Tukey's post hoc tests (B).

To assess the impact of chronic stress on neointimal formation, we used a carotid artery L + C double‐injury model in CD8a^+/+^ mice that had been subjected to 14 consecutive days of chronic stress (Figure 2A). The stress caused markedly increased neointimal hyperplasia in the injured carotid arteries, as demonstrated by a greater neointima/media area ratio, an enlarged neointimal area, and elevated intimal cell counts compared to the non‐stressed L + C controls (Figure 5A,B). Immunofluorescence staining further confirmed that the stress markedly enhanced the infiltration of CD8^+^ T cells into the injured arteries of the CD8a^+/+^ mice (Figure 5C,D), which suggests that chronic stress can accelerate injury‐related CD8^+^ T‐cell infiltration in mice, leading to the facilitation of neointimal hyperplasia. Consistently, the stress also increased the numbers of IFN‐γ^+^/CD8^+^ T cells in the injured arteries of the CD8a^+/+^ mice as compared to the injured alone CD8a^+/+^ mice (Figure 6).

### 3.4 CD8 ^+^ T‐Cell Deficiency Inhibited Neointimal Hyperplasia in Injured Arteries of the Mice Subjected to Chronic Stress

**FIGURE 7:**
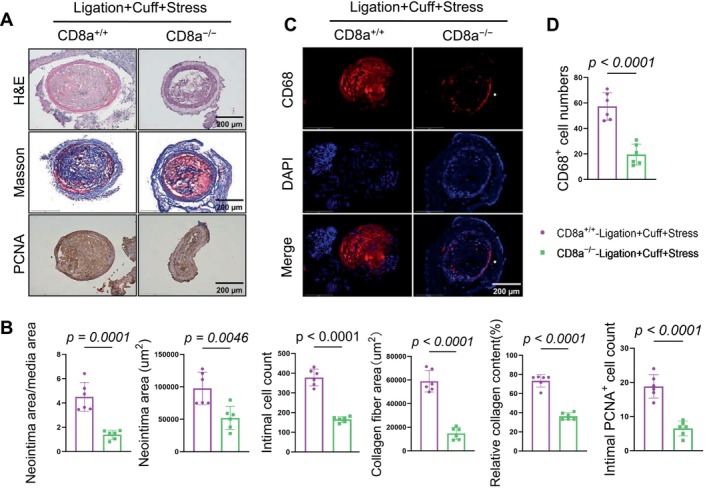
CD8^+^ T‐cell deletion attenuated the stress/L + C–induced carotid artery neointimal hyperplasia. CD8a^+/+^ and CD8a^−/−^ mice that had been subjected to chronic (14‐day) stress plus the L + C injury were subjected to sampling for the histological analyses. (A, B) Representative H&E, Masson's trichrome, and PCNA staining images and combined quantitative data show the neointima/media area ratio, neointimal area, intimal cell count, collagen fiber area, relative collagen content, and number of PCNA^+^ cells in the intima of the two experimental groups. (C, D) Representative immunofluorescence images and quantitative data for the numbers of infiltrated CD68 (*red*) in carotid artery sections of the two experimental groups. Scale bar: 200 μm. Data are mean ± SEM (*n* = 6 for each group). Significance was assessed by a one‐way ANOVA followed by Tukey's post hoc tests (B, D).

To further examine the contribution of CD8^+^ T cells to vascular remodeling related to the combination of injury and chronic stress, we subjected mice of both the CD8a^+/+^ and CD8a^−/−^ genotypes to a double injury followed by 14 days of chronic stress. Compared to the stressed CD8a^+/+^ mice, the CD8a^−/−^ mice exhibited markedly attenuated vascular remodeling, as demonstrated by reduced neointima/media area ratios, a reduced neointimal area, and lower intimal cell numbers (Figure 7A,B). The quantitative data from Masson's trichrome staining showed a marked reduction in the collagen deposition and number of PCNA‐positive proliferating cells in the intima lesions of the CD8a^−/−^ mice. CD8^+^ T‐cell deletion reduced the infiltration of CD68^+^ macrophages in the mice under the stress condition (Figure 7C,D).

**FIGURE 8:**
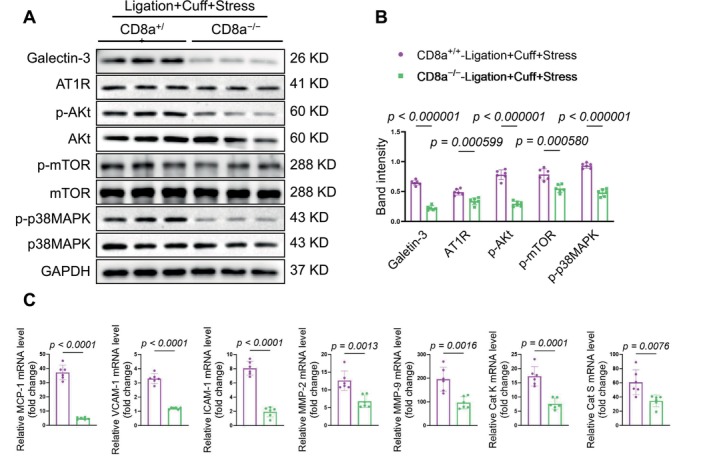
CD8^+^ T‐cell deficiency suppressed the investigated molecular alterations in the carotid arteries in response to the stress plus L + C injury. (A, B) Representative western blot images and combined quantitative data for the levels of galectin3, AT1R, pAkt, pmTOR, and pP38MAPK proteins in the carotid arteries of both experimental groups. (C, D) The qPCR analyses revealed the levels of MCP1, VCAM1, ICAM1, MMP2, MMP9, cathepsin K, and cathepsin S genes. Data are mean ± SEM (*n* = 6/group). Significance was assessed by unpaired Student's *t*‐test (B, C).

The western blot results revealed that the chronic stress produced harmful changes in the levels of galectin‐3, AT1R, p‐Akt, p‐mTOR, and p‐p38MAPK in the injured arteries of the CD8a^+/+^ mice; these changes were reversed by CD8^+^ T‐cell deletion (Figure 8A,B). In a similar manner, the qPCR analysis demonstrated that CD8^+^ T‐cell deletion suppressed the expressions of inflammatory (MCP‐1, ICAM‐1, and VCAM‐1) mRNAs and proteolytic enzyme (MMP‐2, MMP‐9, cathepsin K, and cathepsin S) mRNAs (Figure 8C). Collectively, these observations indicate that CD8^+^ T‐cell deletion provided resistance to the injury via the modulation of inflammation and proliferative signaling in mice under our experimental conditions.

### 3.5 IFN‐γ Deficiency Prevented Stress + Injury‐Related Neointimal Hyperplasia

**FIGURE 9:**
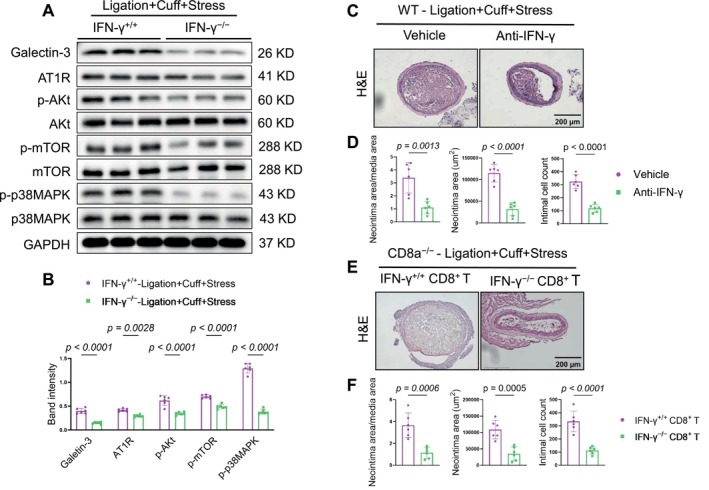
IFN‐γ signaling promoted CD8^+^ T‐cell recruitment and neointimal formation in mice subjected to the 2‐week chronic stress conditions. IFN‐γ^+/+^ and IFN‐γ^−/−^ mice that had received double injuries underwent the chronic stress and were then subjected to sampling for immunoblotting. (A, B) Representative western blot images and quantitative data show the levels of galectin‐3 and AT1R proteins in the carotid arteries of the mice in both experimental groups. IFN‐γ^+/+^ mice that had received double injuries underwent the stress+vehicle intervention or stress+IFN‐γ neutralizing antibody (Anti‐ IFN‐γ) intervention, respectively, for 2 weeks and were then sampled for the histological analysis. (C, D) Representative H&E staining and CD8a immunofluorescence images and quantitative data present the neointima/media area ratio, neointimal area, intimal cell counts, and CD8^+^ T cell numbers in the injured arteries of IFN‐γ^+/+^ mice. CD8a^−/−^ mice that had received double injuries underwent stress plus an adoptive transfer with CD8^+^ T cells isolated from stressed IFN‐γ^+/+^ or stressed IFN‐γ^−/−^ donor mice, respectively for 2 weeks and were then subjected to sampling for the histological analysis. (E, F) Representative H&E and CD8a immunofluorescence and quantitative data show the neointima/media area ratio, neointimal area, and intimal cell numbers in the carotid arteries of both experimental groups. Data are mean ± SEM (*n* = 6/group). Significance was assessed by unpaired Student's *t*‐test (B, D, F).

**FIGURE 10:**
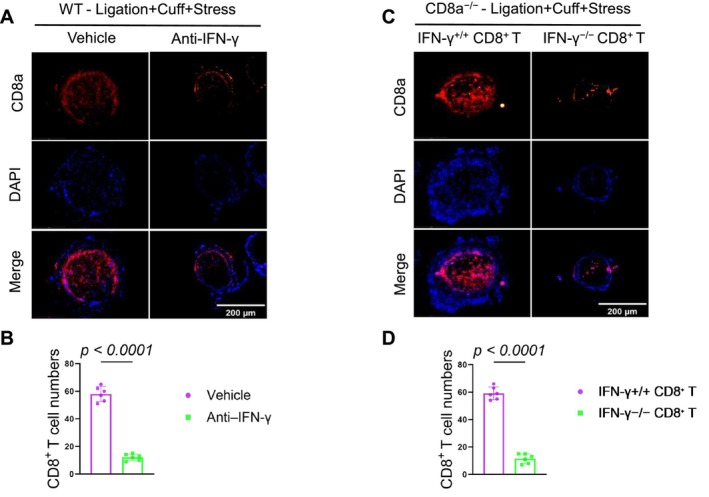
IFN‐γ depletion and deletion reduced CD8a + T‐cell infiltration into the injured carotid arteries of the stressed mice. (A, B) Representative immunofluorescence images and quantitative data for the numbers of infiltrated CD8a (*red*) in injured carotid artery sections of the vehicle and IFN‐γ depletion groups. (A, B) Representative immunofluorescence images and quantitative data for the numbers of infiltrated CD8a (*red*) in injured carotid artery sections of IFN‐γ^+/+^CD8^+^ T‐cell and IFN‐γ^−/−^CD8^+^ T‐cell mice. Scale bar: 200 μm. Data are mean ± SEM (*n* = 6 for each group). Significance was assessed by a one‐way ANOVA followed by Tukey's post hoc tests (B, D).

‐The western blotting assay revealed that the injured arteries of IFNγ^−/−^ mice had decreased levels of galectin‐3, AT1R, p‐Akt, p‐mTOR, and p‐p38MAPK‐ proteins compared to IFNγ^+/+^ mice (Figure 9A,B). Similar to the findings obtained with the CD8a^−/−^ mice, IFN‐γ depletion with its neutralizing antibody resulted in a reduction of neointimal hyperplasia in CD8a^+/+^ mice in response to the 2‐week stress and double injury (Figure 9C,D). Anticipated, IFN‐γ depletion reduced the numbers of CD8^+^ T cells in the injured arteries of the stressed CD8a^+/+^ mice (Figure 10A,B).

Next, to further determine whether CD8^+^ T cell‐derived IFN‐γ plays a role in vascular injury, we adoptively transferred CD8^+^ T cells isolated from stressed IFNγ^+/+^ or IFNγ^−/−^ donor mice into CD8a^−/−^ recipients following stress and injury. The quantitative data of H&E staining and immunofluorescence revealed that compared to the transfer of stressed IFNγ^+/+^‐/CD8^+^ T cells, the transfer of stressed IFNγ^−/−^/CD8^+^ T cells resulted in attenuated CD8^+^ T cell infiltration and remodeling (Figures 9E,F and 10C,D). These findings thus suggest that IFN‐γ is essential for the pro‐remodeling function of CD8^+^ T cells in mice under chronic stress conditions.

### 3.6 The S‐Serum From CD8a ^+/+^ Mice Promoted the Activation of Proliferative Signaling Pathways and Migration in VSMCs

**FIGURE 11:**
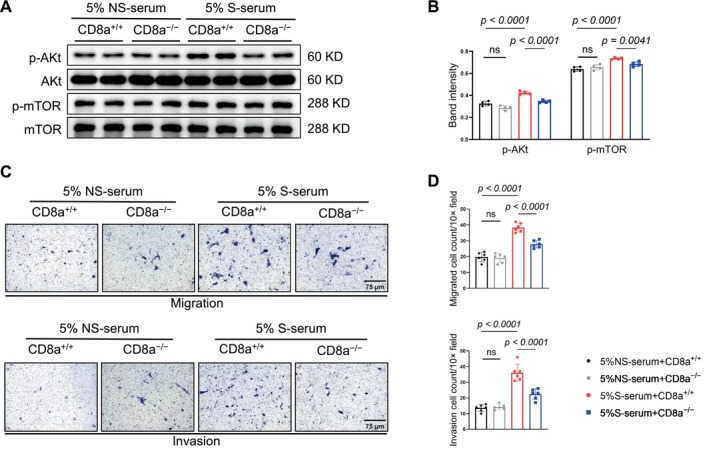
The effects of mouse S‐serum on VSMCs' intracellular signaling and their function. Mouse VSMCs were treated with 5% Non‐serum/CD8a^+/+^, 5% Non‐serum/CD8a^−/−^, 5% S‐serum/CD8a^+/+^, or 5% S‐serum/CD8a^−/−^ for 12 h and then subjected to a western blotting assay. (A, B) Representative western blot images and combined quantitative data show the levels of p‐Akt and p‐mTOR in the four experimental groups (*n* = 4 per group). Here, 5% Non‐serum/CD8a^+/+^, 5% Non‐serum/CD8a^−/−^, 5% S‐serum/CD8a^+/+^, and 5% S‐serum/CD8a^−/−^ were respectively added to the out‐wells of transwells. Mouse VSMCs were seeded onto the inner chambers with serum‐free DMEM and then cultured (for 6 h for the migration assay or overnight for the invasion assay). (C, D) Representative microscopy and quantitative data show the numbers of migrated and invaded VSMCs. Scale bar: 75 μm. Data are mean ± SEM (*n* = 4–6 for each group). Significance was assessed by a one‐way ANOVA followed by Tukey's post hoc tests (B, D).

Finally, to investigate whether S‐serum influences VSMC functions, we treated VSMCs with serum (5%) derived from CD8a^+/+^ or CD8a^−/−^ mice under NS‐serum or S‐serum, respectively. We observed that the phosphorylations of Akt and mTOR were sensitive to the CD8a^+/+^‐S‐serum, and this change was rectified by CD8a^−/−^‐S‐serum (Figure 11A,B). The Transwell migration and invasion assays showed that the CD8a^+/+^‐S‐serum promoted VSMC migration and invasion abilities compared to CD8a^−/−^‐S‐serum (Figure 11C,D). Collectively, these results indicate that the CD8a^+/+^‐S‐serum modulated the migration, invasion, and proliferation of VSMCs, leading to vascular remodeling in the mice under our experimental conditions.

## 4 Discussion

**FIGURE 12:**
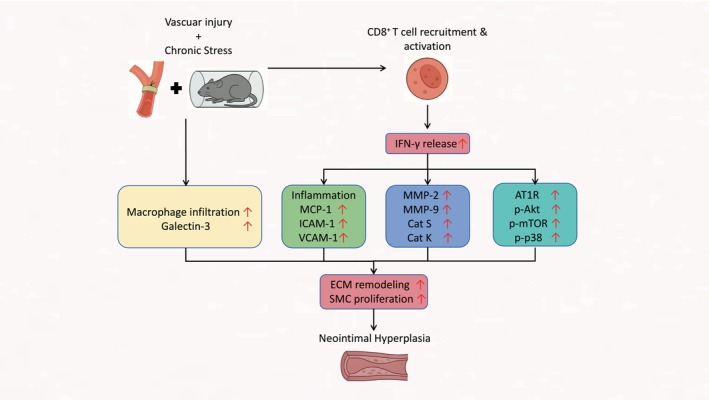
Graphical illustration of the mechanism of vascular remodeling under chronic stress. CD8+ T‐cell functions as an important mediator of injury‐related experimental neointimal hyperplasia via the modulation of inflammation, proteolysis and proliferation that is mediated by the IFN‐γ/AT1R‐galactin‐3 and mTOR/Akt‐p38MAPK axes. MCP‐1, monocyte chemoattractant protein‐1; ICAM‐1, intracellular adhesion molecular‐1; VCAM‐1, vascular cellular adhesion molecular‐1; MMP‐2, matrix metalloproteinase‐2, MMP‐9, matrix metalloproteinase‐9, Cat S, cathepsin S; Cat K, cathepsin K; AT1R, angiotensin type 1 receptor; p‐Akt, protein kinase‐B; p‐mTOR, phospho‐mammalian target of rapamycin; p‐p38, phospho‐p38 mitogen activation protein kinase.

Although immune cell‐mediated tissue remodeling has been an emerging paradigm in the field of ACVDs, there is very limited evidence indicating that adaptive immune cells such as cytotoxic CD8^+^ T cells contribute to chronic stress‐related vascular remodeling and dysfunction after intravascular therapies. In this study we focused on the role(s) of the CD8^+^ T/IFN‐γ axis in injury‐induced neointimal hyperplasia in mice under non‐stress and stress conditions, and the most significant finding is that mice lacking the CD8^+^ T cells/IFN‐γ axis were resistant to L + C injury with or without chronic stress and exhibited less vascular remodeling and neointimal formation. At the cellular and molecular levels, CD8^+^ T‐cell deletion and IFN‐γ depletion were each observed to ameliorate the injured arterial tissue protective changes in the following ways: (i) by decreasing the levels of growth signaling (p‐mTOR, p‐Akt, p‐p38MAPK) proteins; (ii) by reducing the levels of inflammatory (galactin‐3, AT1R, MCP‐1, ICAM‐1, VCAM‐1) and proteolytic enzyme (MMP‐2, MMP‐9, cathepsin S, cathepsin K) genes and/or proteins; and (iii) by reducing the numbers of infiltrated macrophages. The mechanisms underlying the amelioration of injury‐induced neointimal formation in the CD8a^−/−^ mice are schematically represented in Figure 12.

CD8^+^ T‐cell deletion also yielded preventive effects on these injury‐related molecule alterations in mice, even those subjected to chronic stress. Moreover, the data from the IFN‐γ neutralizing and adoptive transfer experiments further confirmed that the lack of the CD8^+^ T cells/IFN‐γ axis produced vasculoprotective actions. Interestingly, the single‐cell RNA sequencing analysis revealed 18 transcriptionally distinct cell clusters, with T cells localized in spatial proximity to endothelial cell and VSMC clusters. The ligation injury resulted in increases in the levels of Ifit1/3, VCAM1, ICAM1, and Ccl7/Cxcl12 genes. The functional enrichment of whole‐tissue DEGs underscored the carotid artery extracellular matrix remodeling, focal adhesion, and proliferation‐related PI3K/Akt activation in response to the ligation injury.

Our in vitro experiments revealed that compared to the 5% S‐serum from the stressed CD8a^+/+^ mice, the 5% S‐serum from the stressed CD8a^−/−^ mice reduced VSMC migration and invasion and reduced the levels of p‐mTOR and p‐Akt proteins in VSMCs. Collectively, these findings suggest that the CD8^+^ T cells/IFN‐γ axis contributed to the injury‐related vascular remodeling and neointimal hyperplasia in the mice with and without the chronic stress conditions.

The ability of the L + C surgery‐induced stress to activate CD8^+^ T cells in the injured carotid artery tissues is likely to have contributed to the deterioration of vascular remodeling and neointimal formation in the mice. Accumulating laboratory and clinical evidence indicates that the host immune system has a pivotal role during tissue remodeling in response to mechanical injury [31, 32]. Recent experimental data highlighted the roles of adaptive immune cells as an essential player in cardiac tissue remodeling [33]. It has been demonstrated that T cells (especially cytotoxic CD8^+^ T cells) negatively modulate the regeneration and remodeling of various organ systems [20, 34, 35]. Our present study revealed that CD8^+^ T‐cell deletion improved the double injury‐induced vascular remodeling and neointimal hyperplasia in mice. The injured carotid arterial tissues of the CD8^−/−^ mice had elevated levels of p‐mTOR, p‐Akt, and p‐p38MAPK proteins. The functional enrichment of whole carotid artery‐tissue DEGs highlighted the ligation‐induced carotid artery ECM remodeling and proliferation‐related PI3K/Akt activation. Because mTOR/Akt‐p38MAKP activation promotes the proliferation of VSMCs in various pathobiologies [36, 37, 38], we propose that in the murine model applied in the present study, CD8^+^ T cells facilitated carotid artery remodeling and neointimal formation in injury states via the activation of proliferative mTOR/Akt‐p38MAKP signaling.

The ability of chronic stress to promote CD8^+^ T‐cell activation is also likely to have accelerated the double injury‐induced vascular remodeling and neointimal hyperplasia in the mice. Chronic psychological stressors caused a deterioration of cardiac fibrosis and dysfunction and atherosclerotic lesion formation in several animal models [28, 39]. Our present results revealed that stress promoted vascular remodeling and neointimal formation in mice, accompanied by intracellular mTOR/Akt‐p38MAKP signaling activation in response to the L + C injury. It was recently reported that IFNγ^−/−^ confers resistance to calcium chloride2‐induced aortic aneurysm formation in mice [19]. We have shown that IFNγ deficiency also ameliorated stress+double injury‐induced carotid artery neointima formation. Moreover, we observed that IFNγ depletion and adoptive transfer with the stressed IFNγ^−/−^/CD8^+^ T cells yielded the same anticipated results concerning the neointimal hyperplasia in the mice under the stress plus double‐injury condition. The CD8^+^ T‐cell/IFN‐γ^−/−^ axis thus functions as an important mediator of injury‐related vascular remodeling and neointimal hyperplasia in the mice under our present experimental stress conditions. This notion was further supported by our cellular experimental observations that the 5% S‐serum from stressed CD8a^−/−^ mice reduced the VSMC migration and invasion abilities and the levels of p‐mTOR and p‐Akt proteins in VSMCs.

T cell‐derived IFN‐γ is harmful to several types of vascular and metabolic diseases, due to immune‐inflammation cross‐talk [19, 35]. We verified the pro‐inflammatory CD8^+^/IFN‐γ axis from multiple perspectives. Both morphological and molecular analyses confirmed that the CD8a^−/−^ mice exhibited significantly reduced collagen deposition and inflammatory cell infiltration compared to the CD8a^+/+^ mice. Immunofluorescence staining revealed that the absence of CD8^+^ T cells resulted in a substantial reduction in the number of CD68^+^ macrophages in the vessel wall, suggesting that CD8^+^ T cells may play a role in mobilizing or activating macrophages. We observed that the L + C injury resulted in increases in the levels of MCP‐1, VCAM‐1, and ICAM‐1 in the injured vascular tissues of the CD8^+/+^ mice both with and without chronic stress conditions, and these changes were markedly reversed in the CD8^−/−^ mice. Comparable vasculoprotection was observed in the stressed IFN‐γ^−/−^ mice, whereas these effects were almost completely diminished by the administration of IFN‐γ. Thus, a reduction of immune‐inflammation cross‐talk by CD8^+^ T‐cell deletion and IFNγ deficiency could represent a common mechanism in the protection of vascular tissues against L + C injury or/and stress.

The ability of proteolytic enzymes (i.e., cathepsins and matrix metalloproteinases [MMPs]) to degrade vascular ECM proteins and growth factors is known to contribute both positively and negatively to vascular remodeling and dysfunction [40, 41]. Our research group's earlier laboratory data demonstrated that MMP‐2 deficiency mitigated injury‐induced experimental neointimal hyperplasia via the modulation of VSMC invasion and apoptosis [42, 43]. In patients who had experienced an acute myocardial infarction, recombinant MMP‐2 induced the shedding of membrane‐bound CD100 from CD8^+^ T cells and the generation of soluble CD100, resulting in an elevation of CD8^+^ T‐cell cytotoxicity toward vascular endothelial cells [44]. Our qPCR results showed that CD8^+^ T‐cell deletion markedly reduced the expressions of MMP‐2/‐9 and CatS/K in the injured artery tissues of mice with and without chronic stress. In other studies, genetic and pharmacological interventions targeting CatS and CatK inhibited experimental injury‐induced neointimal formation via the modulation of VSMC proliferation and invasion abilities in mice under chronic stress conditions [26, 45].

It was reported that (i) the MMP activity within the atherosclerotic lesions of animals was impacted by the deletion of IFN‐γ on CD8^+^ T cells in a murine abdominal aortic aneurysm model, and (ii) this effect was restored by treatment with either recombinant IFN‐γ‐ or IFN‐γ^+/+^‐producing CD8^+^ T cells [46]. Our group recently demonstrated that IFN‐γ stimulates the expression and activities of MMP‐2 and MMP‐9 in vivo and in vitro, leading to aneurysm formation [19]. IFN‐γ has also been shown to modulate CatS in animal pathobiology [47]. Collectively, these observations suggest that CD8^+^ T cells act as a key mediator of vascular remodeling and neointimal hyperplasia via the modulation of IFN‐γ‐mediated proteolysis in mice under our experimental conditions.

Limitations of this study should be addressed. The animal restraint stress model that we used cannot fully mimic the status of human psychological stress. We were unable to investigate whether CD8a^+^ dendritic cells control injury and/or stress‐related neointimal formation via IFN‐γ production and interleukin (IL)‐12‐mediated Th1 differentiation in mice under our experimental conditions. We also could not obtain direct evidence of IFN‐γ‐mediated vascular and inflammatory cellular interactions in the initiation and progression of neointimal formation. In addition, we did not fully explore the cell source of IFN‐γ in vivo. It is noteworthy that although we focused primarily on the role of CD8^+^ T cells in this study, other immune cell subsets (including CD4^+^ T cells, regulatory T cells, B cells, and neutrophils) also play important roles in vascular injury and remodeling. The pro‐inflammatory cytokines IFN‐γ and tumor necrosis factor (TNF)‐α secreted by CD8^+^ T cells also contribute significantly to vascular damage and remodeling. Moreover, CD8^+^ T cells may interact with macrophages in a positive feedback loop, co‐releasing and amplifying pro‐inflammatory factors. Future studies should investigate the interactions between CD8^+^ T cell subsets (e.g., Tc1, Tc2, Tc17) and macrophages (M1/M2) to gain deeper insights into the precise mechanisms through which CD8^+^ T cells regulate vascular injury.

In conclusion, the results of this study underscore the pro‐inflammatory and pro‐remodeling effects of CD8^+^ T cells in vascular injury, particularly in the context of chronic stress. We obtained evidence of CD8^+^ T‐cell facilitation of injury‐related vascular remodeling and neointimal hyperplasia by IFN‐γ‐mediated inflammation, proteolysis, and proliferation in mice with and without chronic stress conditions. Our findings offer new avenues for the development of novel therapeutics targeting a CD8^+^ T‐cell/IFN‐γ axis in injury‐related cardiovascular remodeling and dysfunction in animals under chronic stress conditions. Future research should also focus on targeting CD8^+^ T‐cell activation or on alleviating the effects of chronic stress in order to develop new therapeutic strategies for cardiovascular diseases. Our findings should be validated in other vascular disease models such as atherosclerosis and abdominal aortic aneurysm models, providing a foundation for immune modulation and stress management as part of an integrated therapeutic approach.

## Author Contributions

**Jingyuan Jin:** conceptualization, formal analysis, investigation, methodology, writing – original draft. **Meiling Piao:** investigation, methodology. **Xianji Piao:** investigation, methodology. **Shangzhi Shu:** data curation, methodology. **Longguo Zhao:** methodology, formal analysis. **Zhibo Wang:** methodology, formal analysis. **Xueling Yue:** methodology, formal analysis. **Jinshun Piao:** methodology. **Xianglan Jin:** writing – review and editing. **Lina Hu:** supervision, writing – original draft. **Yongshan Nan:** validation, writing – review and editing. **Xian Wu Cheng:** conceptualization, funding acquisition, project administration, supervision, writing – original draft, writing – review and editing. All authors read and approved the article.

## Disclosure

The authors have nothing to report.

## Ethics Statement

The animal study protocols were approved by the Institutional Animal Care and Use Committees of Yanbian University (protocol no. YD20231212004) and Nagoya University (protocol nos. 30121 and 30068 for the experiments with CTSK^+/+^ and CTSK^−/−^ mice) and were performed in accordance with the Guide for the Care and Use of Laboratory Animals published by the U.S. National Institutes of Health.

## Conflicts of Interest

The authors declare no conflicts of interest.

## Data Availability

The data underlying this study can be shared upon reasonable request to the corresponding author.
